# Four Cases of Poisoning by the Berries of Coriaria Myrtifolia
*The tanners, or myrtle-leaved Sumach; a genus of the Decandria order, belonging to the Diœcia class, and in the natural method ranking under the 54th order Miscellaneæ.—Editors.


**Published:** 1829-04

**Authors:** A. Roux

**Affiliations:** Head Surgeon of the Hôtel-Dieu of Montaubon.


POISONING BY THE BERRIES OF CORIARIA MYRTIFOLIA.

*Four Cases of Poisoning by the Berries of Coriaria Myrtifolia.** By Dr. A. Roux, Head Surgeon of the Hôtel-Dieu of Montaubon.

On the 13th July, 1828, four little girls, named Rose Bertrand, Eulalie and Nancy Chapelle, and Julie Dompeyre, respectively eight, seven, six, and three years and a half old, while strolling among the hedges in the neighbourhood of Montaubon, inadvertently gathered and ate a considerable number of the berries of the Coraria Myrtifolia, being deceived by their resemblance to the fruit of the Rubies scœsius and Fruticosus, (the blackberry and dewberry.) Rose and Eulalie ate fewer berries than their companions: Nancy only thirty, and Julie from eighty to a hundred; according to the account of those who have survived the effects of the poison.

Half an hour after eating the poisonous berries, Julie was affected as follows: a warm pungent sensation in the tongue, the signs of intoxication, the eyes sparkling and rolling constantly about, the countenance livid, convulsions and trismus, and loss of speech, which this patient never once recovered afterwards. The symptoms of poisoning commenced at half-past six P.M., and the little patient was consigned to the care of Dr. Roux about an hour afterwards.

Dr. Roux thus describes her state when he first saw her:

* The tanners, or myrtle-leaved Sumach; a genus of the Decandria order, belonging to the Diœcia class, and in the natural method ranking under the 54th order Miscellaneæ.—Editors.

the face was bloated and livid, the eyes bright and rolling in their sockets, with dilated pupils; she was affected with convulsive motions, of short duration in general, though they continued longer in the limbs of the left side; she had trismus, foaming at the mouth, and flexion of the fingers, as in epileptic patients. The abdomen presented no unusual appearance; and the pulse was not very quick, but rather full.

A large dose of olive-oil was administered to the patient, which was followed by copious vomiting of the deleterious fruit, mixed with thick tenacious mucus; after which, a repetition of the same remedy was ordered, with a table-spoonful of orange-flower water.

At eight o'clock, milk and oil were alternately prescribed; and the patient continues in the same state as at first.

At nine o'clock.—The convulsions last eight or ten minutes at a time, and are separated by nearly equal intervals of repose. The commencement of each paroxysm is preceded by loud groans, five or six times repeated. The belly is becoming tympanitic, but is not tender. The patient bears severe pinching without any sign of pain. The pulse is becoming weaker.—Enemata of milk ordered to be repeatedly injected.

Eleven o'clock.—The head appears to be still more affected. Ten leeches to the legs; continuation of the clysters. The clysters bring away portions of imperfectly digested berries. The pulse is becoming stronger.

Midnight.—The convulsive paroxysms are shorter, and return more speedily.—The clysters as before.

Five o'clock A.M.—Dr. Raynaud visited the patient: he found the pulse small and feeble, and observed the same symptoms as those last described. He ordered that ten leeches should be applied behind the ears, and that the patient should be immersed in a bath.

Eight o'clock A.M.—The disease is in every respect more severe, and the cerebral disorder is particularly increased.

The patient died on the 14th, at half-past ten A.M. The body being immediately examined, the following appearances were observed:

The whole body is stiff, and the fingers still retain the convulsive crook; face pale, the lips livid; the belly very tympanitic and livid; the subcutaneous cellular membrane was every where, except at the feet and anterior part of the tibia, highly vascular. The membranes of the brain were much injected; there was a very small extravasation at the base of the cranium; the brain was healthy; the plexus choroides pale; there was a small quantity of serum in the fourth ventricle; the others were empty, and of a natural appearance. The spinal cord seemed healthy, but its membranes were injected. The heart and pericardium were sound; the left ventricle full of blood. The right lung was hepatized at the posterior part of the lower lobe, and in the centre of this part there were some osseous granulations. On the left side, toward the lower portion of the lung, was a more recent hepatization, which distilled a frothy kind of serum, of the colour of chocolate. In every part this lung was sound, and crepitated when pressed together, but was in general gorged with blood. The œsophagus was inflamed at its cardiac orifice.

The interior of the abdomen was livid like the exterior. The stomach and intestines were greatly distended with gas. The stomach and large intestines externally had the colour of grey satin; the small intestines and omentum presented here and there small patches of red; but the mesentery was studded with glands decidedly redder than the intestine to which they corresponded. The inner surface of the stomach was of its natural colour, except at its greatest extremity, where there was a red spot of the size of half a-crown. Five or six red patches were observed on the internal surface of the intestine; and about twenty lumbrici, of various dimensions, near the middle of the small intestines.

Julie had always enjoyed excellent health: she did not readily take cold, was never subject to colic, her respiration was free and natural; she ate heartily, and at different periods had received blows on the head.

Nancy Chapelle swallowed about thirty berries of sumach, and in three quarters of an hour was affected with headach, colic, and convulsion, sometimes resembling tetanus, locked jaw, foaming at the mouth, and rolling about of the eyes. She took a grain of tartar emetic in olive oil and milk, which caused her to vomit up about twenty of the berries.

Eulalie Chapelle ate about twenty of the same berries. About an hour afterwards she was seized with a hot pungent sensation in the tongue, with headach, and trembling of the limbs. Milk and oil were prescribed, which caused her to vomit up a part of the poisonous fruit.

After the cessation of the first effects of the poison, Nancy and Eulalie remained during four hours in a comatose state, for which no remedy was administered. They slept the remainder of the night, and awoke in the morning perfectly well.

Rose Bertrand took only about fifteen of these berries, and was affected, within an hour afterwards, with headach, colic, and an acute pain at the epigastrium. She swallowed large doses of milk and oil, which did not, however, occasion immediate vomiting, and she afterwards took a light supper. About six in the morning she vomited a great deal, yet it was impossible to distinguish the smallest atom of the fatal berries among that which her stomach threw off. On the following day she was well.

Fortunately, poisoning with the berries of sumach is of very rare occurrence, and Dr. Roux affirms that there are only three other well-attested cases upon record: two related by Sauvages Delacroix, (Académie Royale des Sciences, 1739,) and another by M. Peiyade, physician to the army of the Pyrenees, in 1811. M. Sauvages informs us that, in September 1731, a child happening to eat some of the berries of the coriaria myrtifolia, was soon afterwards, on returning home, suddenly seized with epileptic fits, which were so violent that she died the next day, in spite of all the remedies which are commonly used in that complaint. The following year, in the same month, a robust labouring man, about forty years old, being extremely thirsty, had the rashness to swallow about fifteen of these berries. In half an hour he was seized with one or two paroxysms of epilepsy, for which he was bled; but, as these paroxysms became more frequent, he was brought to the Hôtel Dieu, where Sauvages, on visiting him, found him in one of those paroxysms above described, without any signs of consciousness, of a livid complexion, and in constant danger of falling out of bed if left alone. An emetic was given him in the interval of the fits, which brought up eight or nine of the said berries; but the same evening he expired, during an attack of the fifteenth paroxysm. The body was examined with care, but nothing could be discovered in the brain or stomach, or indeed any where else, which could be fairly considered the cause of his death, excepting that the stomach still contained five or six berries. M. Sauvages concludes by remarking that he had endeavoured to ascertain the *modus operandi* of this poisonous shrub, but totally without success.

The account which M. Peiyade gives on this subject is analogous to that of Sauvages, but is the result of greater experience. Ten young soldiers in Catalonia ate some berries of the coriaria myrtifolia, and were not brought to him until two of them were already dead, and the remainder in a comatose state. He forthwith administered an emetic, which brought off the stomach some indigested berries. He afterwards gave them acidulated drinks, and employed friction and blisters; and, to alleviate the colic, he prescribed mucilaginous substances.

Two experiments in regard to these berries have been made on the lower animals. The first subject of these investigations was a rabbit, who had swallowed four drachms of the extract of this fruit without exhibiting any visible effect from it whatsoever. A puppy was made the subject of a similar experiment, and it showed no signs of disturbance either in the cerebral or digestive functions. Neither the puppy nor the rabbit evinced any symptoms of poison.

Dr. Roux, on the authority of his own personal experience, and that of the well-attested facts which, together with his own, compose the present article, ventures to draw the following conclusions:

1. That the berries of the coriaria myrtifolia are poison to the human species.

2. That the deleterious principle exerts its influence chiefly on the brain and the spinal cord.

3. That a very small quantity only is sufficient to destroy life.

4. But, notwithstanding, there are circumstances, as in the case of a full stomach, for example, when the dose must be very large to prove fatal.

5. That the most dangerous time for the patient is during the violence of the convulsions.

6. That, when coma supervenes, the danger is nearly over.

7. That poison acts in the same way as the acrid narcotics do.

Dr. Roux fancies that his two penultimate inferences may at first sight appear untenable to some members of the profession; but he defends them by reminding us that, in both the instances noticed by Sauvages, the patient died during the acute stage; that, of the ten soldiers mentioned by Peiyade, only two died, and they died during the acute stage; and that Julie Dompeyre likewise died at that period of the disorder. While, on the contrary, the remaining eight soldiers treated by M. Peiyade were comatose, and recovered; that Nancy and Eulalie Chapelle, who became comatose, also recovered. Dr. Roux, therefore, thinks that his conclusions are warranted and supported by all our present information on the subject.

Dr. Roux thinks that until, by the aid of vegetable chemistry, we discover the nature of the venenose principle which lies concealed in the berries of the coriaria myrtifolia (cases of poisoning by this substance being very rare), it will be impossible to establish its treatment on scientific principles; but that we must at present content ourselves with combating merely the most prominent symptoms, and attend to those evident indications upon which there can exist no difference of opinion. Our first object should, therefore, be to evacuate the contents of the stomach. He recommends the employment of acidulated potions on the first knowledge of the occurrence; and the cautious use of mild opiates as soon as the abdomen is becoming tympanitic. Oily and mucilaginous clysters, emollient fomentations, should also be used; and potions likewise, of similar qualities. Congestion in any organ demands vascular depletion, either local or derivative. The judicious use of blisters, and friction of the limbs, either with or without the aid of embrocations, might too, if varied according to circumstances, be ranked as means for opposing the further effects of this poison.

The berries of the myrtle-leaved sumach are not its only deleterious parts; the leaves and stem likewise possess deadly properties. The young sprouts always produce temporary intoxication in those animals which crop them: nay, more, when they feed immoderately off them, their death from this cause is of no uncommon occurrence.

Although Sauvages, confiding in the result of his own experiments, positively asserts the contrary to all this, yet Dr. Roux's opinions are not in the least changed by such an authority, since he informs us that he has frequently seen sheep go into convulsions, and die, from eating the stems only of the sumach. Besides, it is well known that in the south of France, where this shrub is common, the shepherds, who are well acquainted with its properties, as soon as they discover that one of their flock has eaten of its branches merely, drive it from pasture, and endeavour to make it vomit.

---

